# Editorial: Role of Glial Cells of the Central and Peripheral Nervous System in the Pathogenesis of Neurodegenerative Disorders

**DOI:** 10.3389/fnagi.2022.920861

**Published:** 2022-05-04

**Authors:** Rui Wang, Haigang Ren, Yongjing Gao, Guanghui Wang

**Affiliations:** ^1^Center of Translational Medicine, The First People's Hospital of Taicang, Taicang Affiliated Hospital of Soochow University, Suzhou, China; ^2^Laboratory of Molecular Neuropathology, Jiangsu Key Laboratory of Neuropsychiatric Diseases and College of Pharmaceutical Sciences, Soochow University, Suzhou, China; ^3^Institute of Pain Medicine, Institute of Nautical Medicine, Nantong University, Nantong, China

**Keywords:** microglia, astrocyte, neuroinflammation, neurodegenerative diseases, central nervous system

Glial cells are critical to maintain brain homeostasis by multiple ways, including neuronal support and immunological defense in the development of the central nervous system (CNS) and in the peripheral nervous system (PNS). However, glial cells show progressive dysfunction and damage neurons in diseases, especially in neurodegenerative diseases (NDs) (Giovannoni and Quintana, 2020). Mutations of genes *CD33*, triggering receptor of myeloid cells 2 (*TREM2*), apolipoprotein E (*APOE), GBA1* and *GRN* that are mainly expressed in glial cells have been identified as various AD and PD risk factors in genome-wide association studies (GWAS), sparking insights into shed light on the roles of glial cells in pathogenesis of NDs (Bartels et al., 2020; Lewcock et al., 2020). Moreover, single-cell sequencing analyses provide clearer clues for the understanding of the temporal and spatial heterogeneity of glial cells during the progression of NDs (Colonna and Brioschi, 2020). The manuscripts in this Research Topic focuses on the roles of glial cells in the pathogenesis of NDs in the CNS and PNS. We highlight three specific themes in this topic: (1) the contributions of glia-associated neuroinflammation to the diseases; (2) the roles of the interactions between glial cells and neurons in the diseases; (3) the glia-based therapeutics through the modification of glial activation for the disease treatments.

In NDs, glia-mediated neurodegeneration involves multiple pathways, including glial activation, neuronal damage by different signaling, and immune cell response. Su and Zhou reviewed the recent studies about α-synuclein (α-syn)-induced neuroinflammation in the pathogenesis of PD. They described the signalings of microglia upon α-syn stimulation and the contributions of microglia on the transmission of α-syn pathology. Moreover, they discussed the effects of α-syn on T cells and detailed the subtypes of T cells, either inflammatory or anti-inflammatory in response to α-syn, which indicate the involvements of the autoimmune and adaptive immune responses in PD. It is well known that pattern recognition receptors (PRRs), including toll-like receptors (TLRs), are important for initiating the activation of microglia in response to extracellular stimuli (Colonna and Butovsky, 2017). Gu et al. comprehensively reviewed the roles of G protein-coupled receptors (GPCRs) in microglial activation and their potentials as the therapeutic targets in PD. They discussed the mechanisms of different types of GPCRs in microglial activation and the correlations to the progression of PD. Moreover, they also summarized the mechanisms of the PRRs such as TLRs and NOD-like receptors mediated microglia-associated neuroinflammation in PD. Mitochondria are important organelles that function in the degeneration in neurons and the activation in glia. Rahman and Suk discussed the alteration of mitochondrial dynamics in astrocyte activation, which links the mitochondria, astrocytes and neurodegeneration. Using a spontaneous aging mouse strain and an AD mouse model, Molina-Martinez et al. found that aging and AD promote inflammatory gene expressions in hippocampus, suggesting an increased inflammatory response in aging and AD. By analyses of the spatiotemporal specific co-expression networks in AD, Guo et al. found that more microglial and astrocyte genes are enriched than neurons, further suggesting an involvement of glia in AD.

Accumulating evidence suggests that the communications between glial cells and neurons play important role in the regulation of signal transductions and immune responses in the CNS and PNS. Lana et al. reviewed the roles of interactions among microglia, astrocytes and neurons in the hippocampus. They described the changes of the morphology and functions of glial cells during aging and acute inflammation and discussed how activated microglia and astrocytes interact each other to maintain brain homeostasis during neuronal apoptosis in hippocampus. Under pathological conditions, the release of pro-inflammatory factors from microglia and astrocytes influences glial phagocytosis and damages neurons, which aggravates neuroinflammation. In addition to the production of inflammatory factors by glial cells, the communications between glia and neurons can be mediated by the extracellular vesicles (EVs). Li et al. comprehensively reviewed the different effects of glia-derived EVs on the pathogenesis of NDs. They discussed the beneficial and/or the detrimental roles of EVs secreted by glial cells in NDs. They described the roles of glial EVs in the functional regulation through the glia-glia or glia-neuron transmission of key mediators including miRNA, molecular chaperones, signaling and inflammatory components under the physiological and pathological conditions. They also discussed the potentials of EVs in biomarker development and in therapeutic application in diseases.

To date, the studies on the mechanisms of neurodegeneration provide great insights on the pathogenesis of NDs, therapeutic strategies to slow down the progression of NDs have not yet been succeeded. Based on the importance of glial cells in the pathogenesis of NDs, it is promising to develop therapeutic strategies for modulating the functions of glial cells. Brown and St George-Hyslop reviewed the effects of soluble TREM2 (sTREM2) on the functions of microglia and the inhibition of Aβ aggregation. sTREM2 is secreted by microglia and can activate microglia. It also binds to Aβ to repress Aβ aggregation, which protects against the amyloid plaques in AD animal models. Wang Y. et al. discussed current regenerative strategies with reprogramming astrocytes to functional neurons to replace the loss of neurons in NDs. They summarized the key regulators in regenerative strategies to achieve astrocyte-to-neuron reprogramming in NDs and discussed the advantages and difficulties in regenerative strategy therapies *in vivo*. Interestingly, Kim et al. identified that the sulfonylurea drug gliquidone, a FDA approved drug for the treatment of type 2 diabetes, has strongly inhibitory effects on LPS-induced microglial activation *in vivo* and *in vitro*. Gliquidone blocks LPS-induced inflammasome activation in microglia, suggesting that it holds promise in the treatment of inflammation in NDs.

In summary, this Research Topic summarizes the diverse roles of glia in the pathogenesis of NDs and discusses the molecular mechanisms of glia-associated NDs in response to the genetic and environmental factors.

## Author Contributions

RW drafted the manuscript. HR and YG provided suggestions. GW revised and finalized the manuscript. All authors contributed to the article and approved the submitted version.

## Funding

This work was supported by the National Natural Science Foundation of China (Nos. 32070970 and 32170987).

## Conflict of Interest

The authors declare that the research was conducted in the absence of any commercial or financial relationships that could be construed as a potential conflict of interest.

## Publisher's Note

All claims expressed in this article are solely those of the authors and do not necessarily represent those of their affiliated organizations, or those of the publisher, the editors and the reviewers. Any product that may be evaluated in this article, or claim that may be made by its manufacturer, is not guaranteed or endorsed by the publisher.
